# MicroRNAs: New Insight in Modulating Follicular Atresia: A Review

**DOI:** 10.3390/ijms18020333

**Published:** 2017-02-09

**Authors:** Tesfaye Worku, Zia Ur Rehman, Hira Sajjad Talpur, Dinesh Bhattarai, Farman Ullah, Ngabu Malobi, Tesfaye Kebede, Liguo Yang

**Affiliations:** 1 Key Laboratory of Agricultural Animal Genetics, Breeding and Reproduction, Education Ministry of China, College of Animal Science and Technology, Huazhong Agricultural University, Wuhan 430070, China; wtesfaye68@yahoo.com (T.W.); drzia80@aup.edu.pk (Z.U.R.); drhirasajjad@gmail.com (H.S.T.); bhattaraidinesh22@gmail.com (D.B.); farman_aup@yahoo.com (F.U.); 2 School of Veterinary Medicine, Wollega University, P.O. Box 395, Nekemte, Ethiopia; 3 State Key Laboratory of Agricultural Microbiology, Education Ministry of China, College of Veterinary Medicine Huazhong Agricultural University, Wuhan 430070, China; malobi2003@yahoo.com; 4 Departments of Animal and Aquaculture Sciences, Norwegian University of Life Sciences, P.O. Box 5003, 1432 Ås, Norway; tesfaye.kebede.belay@nmbu.no

**Keywords:** apoptosis, follicular atresia, granulosa cells, microRNA

## Abstract

Our understanding of the post-transcriptional mechanisms involved in follicular atresia is limited; however, an important development has been made in understanding the biological regulatory networks responsible for mediating follicular atresia. MicroRNAs have come to be seen as a key regulatory actor in determining cell fate in a wide range of tissues in normal and pathological processes. Profiling studies of miRNAs during follicular atresia and development have identified several putative miRNAs enriched in apoptosis signaling pathways. Subsequent in vitro and/or in vivo studies of granulosa cells have elucidated the functional role of some miRNAs along with their molecular pathways. In particular, the regulatory roles of some miRNAs have been consistently observed during studies of follicular cellular apoptosis. Continued work should gradually lead to better understanding of the role of miRNAs in this field. Ultimately, we expect this understanding will have substantial benefits for fertility management at both the in vivo or/and in vitro levels. The stable nature of miRNA holds remarkable promise in clinical use as a diagnostic tool and in reproductive medicine to solve the ever-increasing fertility problem. In this review, we summarize current knowledge of the involvement of miRNAs in follicular atresia, discuss the challenges for further work and pinpoint areas for future research.

## 1. Introduction

During the development and maturation of the mammalian ovary, a highly complicated, spontaneous phenomenon known as follicular atresia takes place which gets rid of follicles as well as oocytes. This process occurs consecutively in a dictated way from the time of fetal ovary development until the end of reproductive life. It occurs in the course of both pre-and post-natal ovary development and after birth during the reproductive life, leaving capacity for the future maturation and ovulation of oocytes. However, the rate at which germ cells and follicles are affected varies by developmental stage, with the highest proportion of atresia occurring towards the year of menopause [1,2,3,4,5,6,7,8]. It is estimated that 99.9% of oocytes are lost as a result of atresia [1,8]. The fundamental reason why such a large proportion of oocytes are lost during adult and fetal life is still unclear. However, owing to maturing follicles, atresia is believed to be responsible for the limited lifetime of the oocyte and the changing level of hormones.

MicroRNAs (miRNAs), a kind of non-coding RNA molecule (20–24 nucleotides long), are known to negatively regulate gene expression either by translational suppression or by destabilizing mRNA molecules [9,10,11]. Huge numbers of protein coding genes are thought to be regulated by miRNAs, in a process whereby individual miRNAs target multiple genes and an individual gene is targeted by multiple miRNAs [12,13]. Currently, large numbers of miRNAs have been identified and characterized in different species of mammalians’ reproductive tissues and cells [13,14,15,16,17,18,19]. They are implicated in various regulatory roles of cellular physiological events including oocyte maturation, apoptosis, proliferation, differentiation, development and biogenesis [17,20,21,22,23,24,25,26,27,28,29,30].

Follicular atresia is mainly attributed to granulosa cell (GC) apoptosis, which surrounds oocytes. Several earlier studies have verified that GC apoptosis plays a pivotal role in follicular atresia and a complex array of molecular mechanisms and regulatory networks are involved in the modulation of GC apoptosis caused by extrinsic and intrinsic factors [7,31,32,33,34,35,36,37,38,39,40]. To date, microarray profiling studies of miRNAs in ovarian tissues and cells have identified large numbers of differentially expressed miRNAs during various follicular developmental processes such as primordial development [18], steroidogenesis [41,42,43], luteal development [16,44], and atresia [45,46,47]. In subsequent target prediction and functional enrichment analysis undertaken to understand their functional involvement, some miRNAs have been categorized in apoptotic pathways. In addition, several functional studies have identified the apoptotic regulatory role of miRNAs together with their target genes and signaling pathways during granulosa cell apoptosis.

Recently, it has become clear that a number of miRNAs control follicular atresia through their target genes and different signaling pathways, thereby modulating GC apoptosis [48,49,50,51,52,53]. This work provides new insights to further understand the mechanism of follicular atresia. Previously, the transcriptional regulation of genes during granulosa cell apoptosis, which is responsible for follicular atresia, had been only briefly defined. Hence, the regulatory mechanism of post-transcriptional regulation needs to be investigated and made available. Given the complexity of follicular atresia processes, miRNAs are thought to play a central role in ovarian follicular apoptosis. Therefore, in this review, we pinpoint the current understanding, challenges and perspective of miRNA research with respect to follicular atresia and/or GC apoptosis.

## 2. Profiling of miRNAs during Follicular Atresia

In attempting to understand and identify the specific physiological role of miRNAs at specific developmental stages, a few profiling studies have reported differential expression of miRNAs during a specific follicular atresia stage. These include profiling studies conducted in mammals such as the cow [47] and pig [46,54]. A total of 1133 miRNAs have been identified at various follicular atresia stages using microarrays in porcine ovaries [46], and the highest numbers of miRNAs were identified in progressively atretic follicles, with lower numbers of miRNAs identified in early atretic and healthy follicles. Of those, 200 miRNAs were found to be differentially expressed in healthy vs. early atretic vs. progressively atretic follicles. Among these, 23 miRNAs were differentially expressed between healthy and early atretic follicles, indicating their importance in the initiation of follicular atresia. However, localization of these miRNAs needs to be determined for better understanding of the dynamic change of miRNAs within the follicular compartment. In a similar study, bioinformatics and target prediction analysis revealed that five miRNAs (miR-R26b, miR-1826, miR-1308, miR-936 and let-7a) are functionally enriched to target genes which are known to be involved in apoptotic pathways; however, this study needs to be experimentally validated [46].

In another study, it was reported that as many as 57 miRNAs were found, by employing a microarray approach, to be differentially expressed between large atretic and large healthy follicles in bovine ovarian tissue [47]. Among these, miR-142, miR-21, miR-150, miR-21, miR-409a and miR-378 were abundantly expressed in large atretic follicles. Furthermore, miR-378, miR-409a and miR-21 are commonly studied [14,43,44,55,56]; for instance, the role of miR-21 is well characterized in bovines [14] and mice [57] as survival molecules of GCs. Among miRNA families, the profile of the let-7 (lethal-7) miRNA family has been explored across different stages of follicular atresia and it was observed that the expression levels of all let-7 family members were found to be downregulated in healthy and early atretic follicles, while let-7g was highly upregulated in progressively atretic follicles [54]. The details of the functional studies for let-7g are briefly described in the subsequent section.

**Figure 1:**
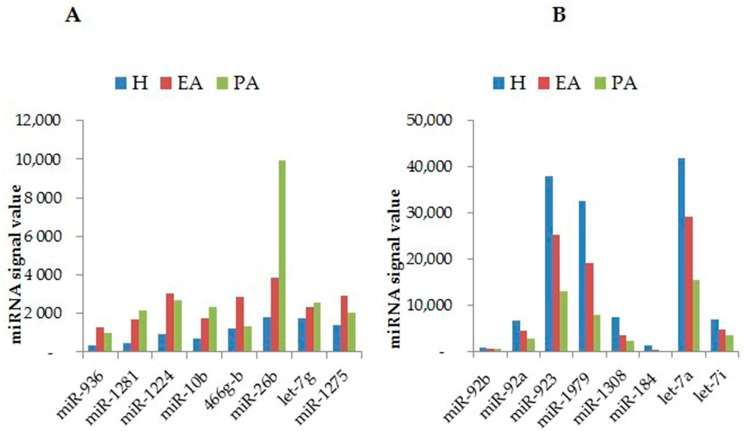
Differential expression of some miRNAs during follicular atresia in pigs. The expression profiles of miRNAs whose expression is upregulated during follicular atresia (**A**); and miRNAs whose expression is downregulated during follicular atresia (**B**) are shown. H, healthy; EA, early atretic; PA, progressively atretic modified from [45,46].

Figure 1 depicts miRNAs which are currently known to be differentially expressed in the porcine ovary during follicular atresia. Of the miRNAs shown in Figure 1, the specific functional roles of miR-26b and let-7g in follicular atresia are summarized in the next section; in contrast, the regulatory roles of the others need to be experimentally confirmed.

## 3. Functional Roles of miRNAs during Follicular Cellular Apoptosis

A growing body of evidence indicates that the number of studies investigating the roles of miRNAs on ovarian follicular apoptosis is greatly increasing. The regulatory roles of miRNAs in follicular GCs, explored in several studies, are shown in Table 1.

The expression level of intercellular pro-apoptotic protein, B-cell lymphoma 2-associated x protein (BAX), was found to be inhibited in human GCs transfected with the miR-22 precursor [41], opening a new window for further investigation on how miR-22 inhibits ovarian GC apoptosis. In particular, which gene it targets to perform its action needs to be identified. In a separate study undertaken to examine the functional role of miR-22 during follicular atresia processes, it has been shown that the expression level of miR-22 decreased progressively from healthy to early atretic and progressively atretic follicles. At the same time, in vitro treatment of mice GCs with miR-22mimic [52] resulted in repressing apoptosis. Further, it has been shown that the SIRT1 gene was targeted by miR-22 and downregulated, thereby suppressing GC apoptosis in mice. The anti-apoptotic role of miR-22 has also been reported in non-ovarian cells, specifically neural cells, a discovery which reinforces the importance of miR-22 in regulating cellular apoptosis [60].

The let-7 family (lethal-7) miRNAs, found across nine different chromosomes in humans, are among the first discovered and conserved miRNAs across animal species [61,62]. Concomitant with their role, they are known to regulate target genes which are responsible in biological events such as GC apoptosis [50], proliferation [63,64], and pluripotency [65,66]. Let-7g, one of the members of the let-7 family, appears to play a significant role in follicular atresia and other diverse biological processes (for example, angiogenesis, proliferation and differentiation). The expression level of let-7g is significantly increased in porcine atretic follicles [45,46], suggesting its role in GC apoptosis (Figure 1). In a subsequent study, transfection of cultured porcine GCs with let-7g induced GC death and further bioinformatics, in vitro analysis has shown that let-7g mediates GC apoptosis by targeting the transforming growth factor-β type 1 receptor (*TGF-β1*) gene [50].An experiment to profile the let-7 family during follicular atresia processes revealed a significant increase of let-7g expression with the progress of atresia [54], suggesting its regulatory role in atresia. In a further study, the expression levels of anti-apoptotic genes B-cell lymphoma 2 family (*BCL-2*) and myeloid cell leukemia (*MCL-1*) were significantly decreased when let-7g was transfected into porcine GCs, thereby enhancing the apoptosis rate as indicated by flow cytometry. Importantly, the anti-apoptotic effect of let-7g is rooted in its ability to target the mitogen-activated protein kinase kinase kinase (*MAP3K1*) gene, which induces the dephosphorylation of Forkhead box protein O1 (*FOXO1*) and finally promotes apoptosis by increasing the cleavage of casepase-3 and associated apoptotic genes [45].

**Figure 2:**
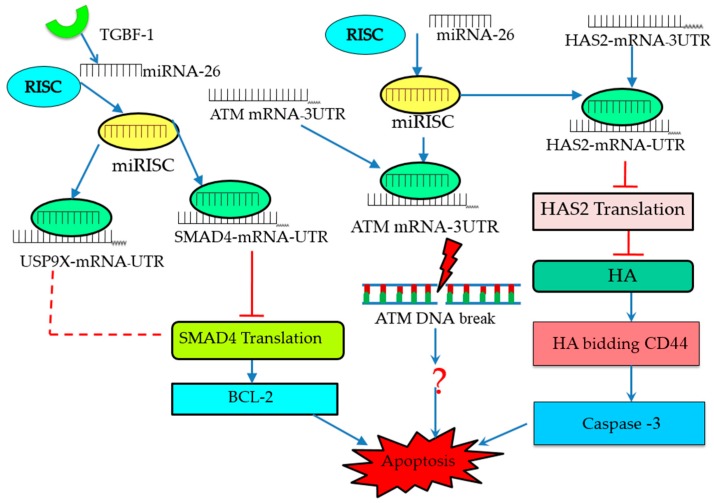
Model of miR-26b–mediated pathways in the regulation of follicular GC (granulosa cell) apoptosis. miR-26b promotes GC apoptosis by activation of BCL2, DNA breaks and caspase-3 through its target genes (SMAD4, ATM and HAS2). miRISC form complex with SMAD4-mRNA and directly block it’s translation, while USP9X form the complex and indirectly inhibit the ubiquitination of SMAD4 and cause apoptosis by activating BCL-2. HAS2-mRNA and miRISC form complex and inhibit the synthesis of HA and cause apoptosis through activating downstream factor, Caspase-3. miRISC, miRNA-induced silencing complex; B-cell lymphoma 2 family, BCL2; SMAD4, Sma and Mad-related protein 4; ATM, ataxia telangiectasia mutated; HA, hyaluronic acid; HAS2, hyaluronan synthase 2; USP9X, ubiquitin specific peptidase 9, X-linked.

In other research, miR-26b was found to be upregulated during porcine follicular atresia, providing evidence that it is developmentally regulated. Further in vitro study showed that miR-26b enhances DNA breaks and GC apoptosis there by targeting (ataxia telangiectasia mutated) (*ATM*) [46]; nevertheless, the downstream effect of miRNA-26b during apoptosis needs to be explored and elucidated (Figure 1). Similarly, another in vitro study reported that miR-26b was found to promote apoptosis by directly and indirectly targeting Sma-and Mad-related protein 4 (*SMAD4*) and ubiquitin specific peptidase 9, X-Linked (USP9X) [48], respectively. Moreover, miR-26b is reported to promote porcine GC apoptosis when it is overexpressed; meanwhile, hyaluronic acid synthase 2 (*HAS2*) has been identified as the target gene of miR-26, for which the apoptotic processes are mediated through the HAS2-CDD44-Caspase-3 pathway [53]. Collectively, these results strongly suggest that miRNA-26b plays a crucial role in GC apoptosis and follicular atresia, while reinforcing the fact that individual miRNAs target multiple genes and involve different patterns of pathways to regulate the follicular apoptosis of GCs (Figure 2).

In one recent study, the involvements of miR-23a and miR-27a in modulating human follicular GC apoptosis were identified both during the normal physiological cycle and disorder. In plasma of premature ovarian failure (POF) patients, miR-23a has been found to decrease X-linked inhibitor of apoptosis protein *(XIAP)* expression (at the mRNA and protein levels) with a simultaneous increase of cleaved caspase-3 protein [17], thereby increasing apoptosis in GCs. Consistent with this, miR-23a and miR-27a have been shown to promote human granulose cell apoptosis in vitro by directly targeting SMAD5 [49]. In this regard, these miRNAs are acting as pro-apoptotic and apoptotic factors in pathological and normal cycling cells, respectively. In two recent in vitro studies, it was shown that miR-92a and miR-34a suppress and promote apoptosis of cultured porcine GCs by directly targeting the Sma-and Mad-related protein 7 (*SMAD7*) and inhibin beta-B (*INHBB*) genes, respectively [48]. Like miR-92a, miR-21 has been found to act as an anti-apoptotic factor as transfection of mural GCs with miR-21targeting locked nucleic acid (LNA) oligonucleotide (miR21-LNA increases cellular apoptosis, presumably suggesting the indispensable role of miR-21 in preventing GCs from undergoing apoptosis [57]. In addition, this was the first study that paved the way for embarking on the study of the role of miRNAs in the field of reproduction. Following transfection of mouse GCs with miR-125a-5p, cleaved caspase-3 was observed. Further investigation of luciferase reporter assays showed that silent mating-type information regulation 2 homologue 1 (*STAT3*) was the functional target of miR-125a-5p [51], suggesting its pro-apoptotic effect.

## 4. Molecular Signaling Pathways Enriched in miRNA-Mediated Follicular Atresia

The existence of miRNAs was discovered about 10 years ago, and since then considerable achievements have been made in understanding the biogenesis pathways, molecular mechanisms, and pathways linked to specific functions of miRNAs during their involvement in various cellular events. These include follicular cellular apoptosis, proliferation, development and pathogenesis. This research has employed in vitro, in vivo and pathway enrichment analyses. Although some extrinsic and intrinsic factors known to regulate apoptosis during follicular atresia in normal and pathological cellular processes have been characterized and established [31], discoveries of new, novel regulatory molecules, including miRNAs, are emerging, revealing additional signaling pathways and compelling researchers to revise our understanding of known pathways.

**Figure 3:**
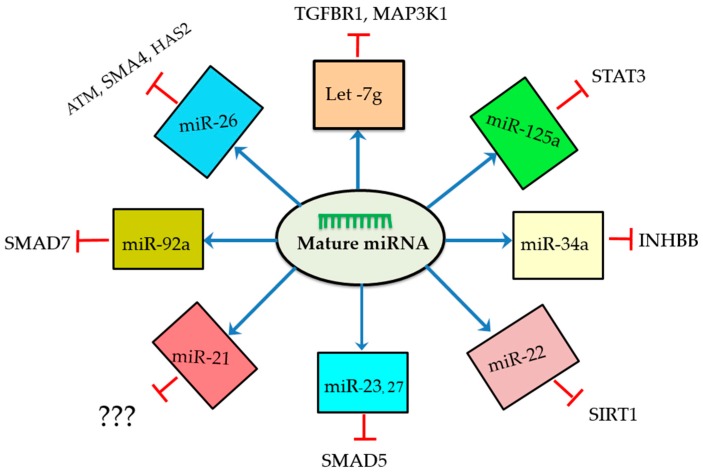
Schematic representation of miRNAs along with their target genes. These miRNAs regulate granulosa cell apoptosis through involving different signaling pathways by negatively regulating their target genes. Mature miRNAs are generated from transcribed miRNA genes and sorted to different signaling pathways and guided to regulate their respective target genes.

We now know that miRNAs control different signaling pathways of GC apoptosis and the ensuing follicular atresia by interacting with the mRNAs of target genes, and they regulate the functions of these genes to the end in follicular atresia processes (Figure 2 and Figure 3). Notably, as shown in Figure 2, mature miRNA molecules form a complex with the RNA-induced silencing complex (RISC) and specifically bind to the 3UTR region of mRNA, leading to their translation repression [67]. The Fas ligand (FasL-Fas) system is one of the well-defined apoptotic signaling pathways known to play an indispensable role during follicular atresia [33,34,38]. The FasL-Fas signaling pathway also modulates the apoptotic role of miR-23a and -27a by downregulating the protein expression level of the target gene smad5 [49]. In several studies, the transforming growth factor beta signaling (TGF-β) pathway appears to control cellular physiological processes associated with mammalian reproduction [68,69,70]. Similarly, the *TGF-β1* signaling pathway has been found to be involved in GC apoptosis and follicular atresia by downregulating miR-26b, in which case *SMAD4* is the target gene (Figure 2 and Figure 3) [59]. Like the *TGF-β1* signaling pathway, the HAS2-HA-CD44-Caspase-3 pathway is well established [71,72] in the regulation of follicular atresia and miR-26 has been shown to promote porcine granulosa apoptosis through HAS2-HA-CD44-Caspase-3 by directly inhibiting the translation of *HAS2*, which is the key target of miR-26 [53]. In another experiment, miR-22/*SIRT1* and miR-92a/*SMAD7* signaling was reported to regulate porcine follicular atresia by targeting *SIRT1* and *SMAD7*, respectively; however, the link with downstream apoptotic genes and proteins remains to be studied [48,52] (Figure 3). Global transcriptome profiling and network analysis of miRNAs in bovine GCs revealed several miRNAs that target apoptosis pathways [14].

## 5. miRNAs-Induced Autophagy Regulates Granulosa Apoptosis

Autophagy is a process responsible for the degradation of cellular components such as proteins and organelles, and it is essential for cell survival. It is triggered by a wide range of stimuli such as lack of nutrients [73] and hypoxia [74], both among autophagy-predisposing factors. Autophagy and apoptosis are interrelated, but the mechanism of interplay has not yet been clearly defined. Accumulating evidence indicates that autophagy and apoptosis are regulated by miRNAs during pathological situations, especially cancer [75]. The correlation of autophagy and apoptosis has been reported in rat granulosa cells, suggesting autophagy may appear to regulate follicular fate [76]. In another study [77], it has also been shown that the interplay between apoptosis and autophagy is mediated by proteins. A recent study conducted by Zhou et al. [78] revealed that autophagy induced by let-7g tends to promote mice GC apoptosis; at the same time, inhibition of autophagy promotes cell survival through inhibiting GC apoptosis by targeting insulin like growth factor 1 *(IGF-1).* In support of this finding, let-7g has also been shown to play an important regulatory role in GC apoptosis via targeting different genes and signaling pathways (Table 1 and Figure 3).

## 6. miRNAs Are Promising Therapeutic Agents and Biomarkers in Follicular Atresia

**Figure 4:**
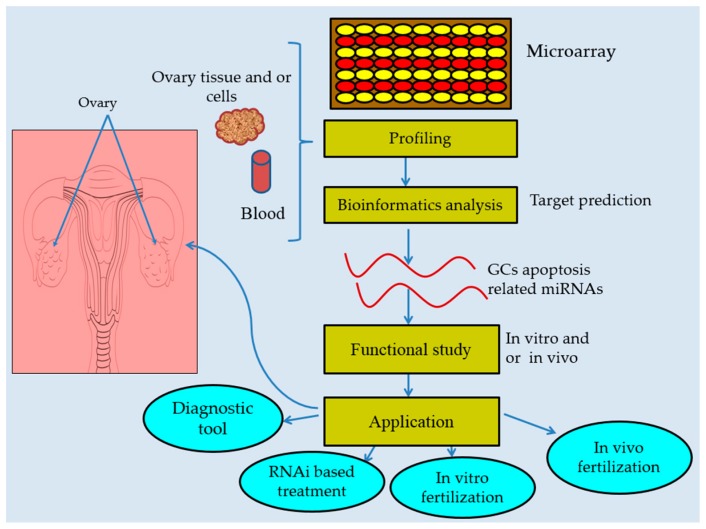
Diagrammatic model for miRNAs characterization and practical relevance during follicular atresia. A cluster of miRNAs have been identified by using microarray techniques and bioinformatics are used to predict their functions. Their specific role is confirmed by in vitro and/or in vivo study for clinical and biological application.

Several miRNAs are reported to be promising therapeutic biomarkers for prognosis and diagnosis of reproduction-related disorders such as ovarian cancer [79,80,81], polycystic ovary syndrome (PCOS) [82], and pregnancy disorder [83,84]. Extracellular miRNAs can serve as powerful tools for diagnosis because of ease of sampling and quantification [85], and of these, blood sample miRNA can serve as a good biomarker tool (Figure 4) as compared to other body fluids [86] owing to its ability to remain intact for a long time and the ease of obtaining samples through minimally invasive techniques. A profiling study of miRNAs revealed differential expression of miR-23a and 27a in blood plasma between premature ovarian failure (POF) patients and normal-cycling women [17]. Further investigation identified miR-23a and miR-27a in promoting GC apoptosis in POF patients whereas miR-23a has been shown to enhance GC apoptosis in the normal physiological process, implying that these miRNAs could be possible biomarkers in diagnosing follicular atresia [17,49]. Likewise, follicular fluid miRNAs such as miR-320a, let-7b and miR-29a have been identified as potential prognosis markers for in vitro fertilization (IVF), suggesting their application in clinical pregnancy management [79] (Figure 4).

Safeguarding follicles from undergoing atresia leads to good quality and competent oocytes, paving a path towards improved fertility. Better understanding of miRNAs-mediated follicular atresia at every junction of atresia processes is imperative to develop effective therapies. Some miRNAs such as miR-26b [46,53,59], miR-21 [57], and let-7g [45,78] are consistently shown to regulate follicular apoptosis by targeting various genes in different species and study models (Table 1 and Figure 3). Furthermore, during follicular atresia, miRNA-26 seems to be involved in different signaling pathways (Figure 2). These miRNAs seem promising for future therapeutic development.

## 7. Concluding Remarks and Future Directions

Despite the important developments made in understanding the specific roles of miRNAs in modulating follicular atresia, there are still gaps in our understanding that need to be explored and filled. Recent profiling studies at specific stages of follicular atresia and genomic-wide miRNA analyses have identified a number of miRNAs associated with follicular atresia and this paves the way for further investigations of the regulatory role of miRNAs during GC apoptosis, which contributes significantly to atresia. For a few miRNAs, the molecular mechanisms and pathways involved during atresia are partially elucidated and have been made available, providing ideas for applications (Figure 4) in clinical settings to mitigate infertility problems. However, a thorough understanding of the full roles of miRNAs, especially from the initiation of atresia to its final stages in the adult and embryonic stages of mammals, is just beginning to evolve and needs to be defined and established. Granulosa cell apoptosis triggers follicular atresia [32], and a high rate of follicular atresia is likely to cause poor fertility and premature ovarian failure (POF), which in turn leads to infertility. Identifying and understanding the molecular mechanisms of miRNAs known to be involved in follicular atresia, under normal and pathological physiological conditions, has versatile clinical and biological potential in reproductive biology (Figure 4).

Notably, the GC apoptotic regulatory role of reproduction-related hormones such as follicle-stimulating hormones (FSH), luteinizing hormone (LH), and progesterone is well characterized and established. Despite this, the association of these hormones with miRNAs during follicular atresia is not yet well studied and made available, except for one study conducted by Kim et al. [87], which reported that the expression profiles of miRNAs are regulated by gonadotropin and vitamin C during follicular growth in vitro. Because the roles of miRNAs are versatile and implicated in many cellular physiological processes, undoubtedly they have post-transcriptional roles in hormone-mediated follicular atresia. This area is an important avenue for future investigation, which would broaden our understanding in this field. The contributions of currently identified and characterized miRNAs are immense for the development of better therapeutics and as biomarker tools for fertility management and improvement in humans and livestock through both in vivo and in vitro fertilization.

## Figures and Tables

**Table 1:** Summary of role miRNAs in granulosa cell (GC) apoptosis: targeted genes, model species, study platform used and changes observed.

Gene Symbol	Functions and Changes during Apoptosis/Atresia	Target Genes	Model Species	Study Types	Reference
let-7g	Inhibits TGB-β1 and induces GC apoptosis	*TGBR1*	pig	In vitro	[50]
	Inhibits MAPK1 induces GC apoptosis	*MAP3K1*	pig	In vitro	[45]
miR-125a	Enhances cleaved caspase-3 and promotes GC apoptosis	*STAT3*	mice	In vitro	[51]
miR-34a	Represses INHBB and promotes GC apoptosis	*INHBB*	pig	In vitro	[58]
miR-22	Suppresses SIRT1 and inhibits apoptosis	*SIRT1*	mice	In vitro	[52]
miR-23a and miR-27a	Increases expression of cleaved caspase-8, cleaved caspase-3, promotes GC apoptosis	*SMAD5*	human	In vitro	[49]
miR-23a	Increased cleaved caspase-3, decrease caspase-3 protein and promote GC apoptosis	XIAP (protein)	human	In vitro	[17]
miR-26b	Increases DNA break, inhibits ATM, and promotes GC apoptosis	*ATM*	pig	In vitro	[46]
	Inhibits BCL-2, suppresses SMAD4, and promotes GC apoptosis	*SMAD4*	pig	In vitro	[59]
	Suppresses HAS2, enhances caspase-3 and promotes GC apoptosis	*HAS2*	pig	In vitro	[53]
miR-92a	Inhibits SMAD7 and anti-apoptotic	*SMAD7*	pig	In vitro	[48]
miR-21	Decreases cleaved caspase 3, inhibits apoptotic	?	mice	in vivo and in vitro	[57]

TGBR1, transforming growth factor-β type 1 receptor; MAP3K1, mitogen-activated protein kinase kinase kinase; STAT3, signal transducer and activator of transcription 3; INHBB, inhibin beta-B; SIRT1, silent mating-type information regulation 2 homologue 1; SMAD5, Sma-and Mad-related protein 5; XIAP, X-linked inhibitor of apoptosis protein ATM, ataxia telangiectasia mutated; SMAD4, Sma-and Mad-related protein 4; HAS2; hyaluronic acid synthase 2; SMAD7, Sma-and Mad -related 7.
